# Pulse-density modulation control of chemical oscillation far from equilibrium in a droplet open-reactor system

**DOI:** 10.1038/ncomms10212

**Published:** 2016-01-20

**Authors:** Haruka Sugiura, Manami Ito, Tomoya Okuaki, Yoshihito Mori, Hiroyuki Kitahata, Masahiro Takinoue

**Affiliations:** 1 Department of Computational Intelligence and Systems Science, Tokyo Institute of Technology, 4259 Nagatsuta-cho, Midori-ku, Yokohama 226-8502, Japan; 2 Department of Chemistry, Faculty of Science, Ochanomizu University, 2-1-1 Ohtsuka, Bunkyo-ku, Tokyo 112-8610, Japan; 3 Department of Physics, Graduate School of Science, Chiba University, 1-33 Yayoi-cho, Inage-ku, Chiba 263-8522, Japan; 4 PRESTO, Japan Science and Technology Agency (JST), 4-1-8 Honcho Kawaguchi, Saitama 332-0012, Japan

## Abstract

The design, construction and control of artificial self-organized systems modelled on dynamical behaviours of living systems are important issues in biologically inspired engineering. Such systems are usually based on complex reaction dynamics far from equilibrium; therefore, the control of non-equilibrium conditions is required. Here we report a droplet open-reactor system, based on droplet fusion and fission, that achieves dynamical control over chemical fluxes into/out of the reactor for chemical reactions far from equilibrium. We mathematically reveal that the control mechanism is formulated as pulse-density modulation control of the fusion–fission timing. We produce the droplet open-reactor system using microfluidic technologies and then perform external control and autonomous feedback control over autocatalytic chemical oscillation reactions far from equilibrium. We believe that this system will be valuable for the dynamical control over self-organized phenomena far from equilibrium in chemical and biomedical studies.

## Abstract (web summary)

Biological systems typically operate at conditions far from chemical equilibrium. Here, the authors model and develop a microfluidic reactor allowing control over time-variable supply and dissipation of chemicals by droplet fusion and fission, allowing non-equilibrium chemical reactions to be regulated.

## Graphical abstract

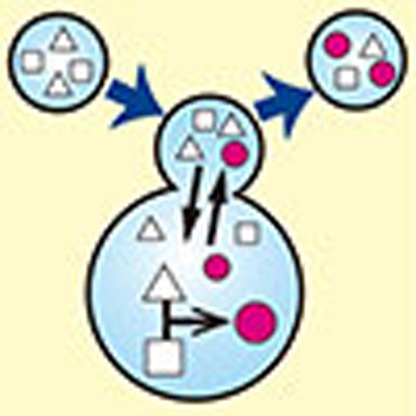

Living systems are achieved by complex chemical reaction dynamics far from equilibrium, such as gene expression networks, signalling networks, metabolic circuits and neural networks. The design, construction and control of artificial bio-inspired self-organized phenomena remain challenging in a wide range of science and engineering fields, such as the use of synthetic biology for understanding life^1^, the fabrication of bio-inspired nano/microscale autonomous artificial systems^2^ and the synthesis of dynamical microscale materials^3^. Recently, micrometre-sized reaction systems modelled on cellular systems have been actively studied, including chemical and biological reactions in microcompartments^4^,^5^,^6^,^7^,^8^,^9^,^10^,^11^,^12^,^13^,^14^,^15^,^16^,^17^,^18^,^19^ and microfluidic devices^20^,^21^,^22^, and artificial multicellular interactions^23^,^24^,^25^.

Chemically open systems with well-controlled chemical fluxes into/out of the systems (that is, supply and dissipation of chemicals) are essential for complex chemical reactions far from equilibrium, to eliminate the increasing entropy in such systems^26^. The construction of microreactors with chemical fluxes is therefore necessary in microscale bio-inspired engineering. Open microreactors, such as semipermeable microcapsules^7^,^15^, liposomes with nanopore proteins^9^,^25^ and on-chip DNA compartment reactors^22^, use passive substrate diffusion to generate chemical fluxes. In contrast, annular microchannels with valves and peristaltic mixers^20^,^21^ produce chemical fluxes by the mechanical injection of solutions. Thus, the use of microreactors to generate chemical dynamics, including chemical oscillations based on constant chemical fluxes, has been successful. However, the time-variable chemical fluxes required for the external control and the feedback control depending on environment and inner reaction states, as in the case of living systems, have never been achieved in microscale systems. The development of useful and robust methods for the precise control of time-variable chemical fluxes is thus an important issue in microscale bio-inspired engineering.

In this paper, we report a microfluidic method that can control time-variable chemical fluxes into/out of a microreactor (Fig. 1). Our method is inspired by the universal molecular transportation systems in cells, which is based on vesicular fusion and fission observed in endo- and exo-cytotic processes, organellar vesicular transportation, and viral infection and budding via envelope. For example, a macrophage cell continually ingests and secretes solutions amounting to 25% of its volume each hour, while its own volume remains constant^27^. Similarly, our method achieves sustained chemical fluxes based on the repeated fusion and fission of microdroplets whose volumes remain constant. In this work, we use water-in-oil (W/O) microdroplets as microreactors. Droplet-based microfluidics enables rapid-response (for example, electrical and magnetic) manipulation of fluids without microfluidic mechanical components, such as valves. We show that the droplet open-reactor system is electrically controlled by the pulse-density modulation of fusion–fission timing, which enables precise control over time-variable chemical fluxes, including external control and autonomous feedback control. We believe that this system will facilitate innovations in chemical and biomedical studies in terms of the dynamical control of self-organized phenomena far from equilibrium.

## Results

### Mathematical analyses of the droplet open-reactor system

**Figure 1:**
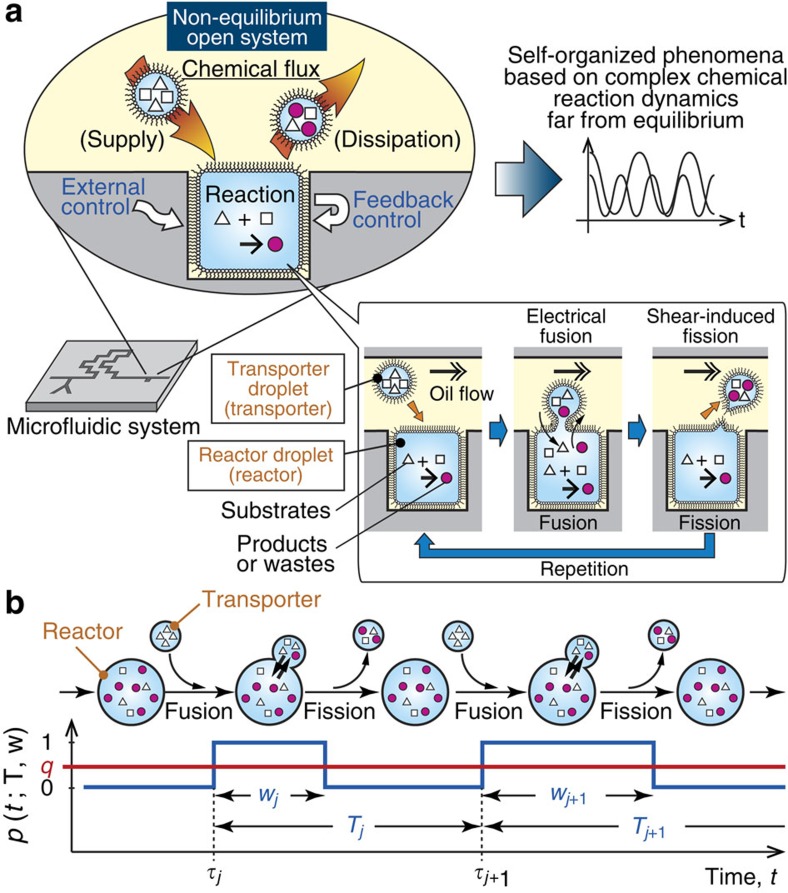
Schematic diagram of chemical reactions far from equilibrium in a droplet open-reactor system controlled by pulse-density modulation. (**a**) In the droplet open-reactor system, the supply of substrates and the dissipation of products/wastes into/out of the reaction system are sustained, inducing self-organized phenomena based on complex chemical reaction dynamics far from equilibrium. The chemical reactions in the droplet open-reactor system are dynamically varied based on external control and feedback control. The droplet open-reactor system is based on the repeated fusion and fission of droplets. (**b**) A fusion–fission process and the pulse-density modulation concept. *T*_*j*_ and *w*_*j*_ are the interval and duration of *j*-th fusion–fission event, respectively. *p*(*t*; **T**, **w**) is a square pulse-train function used to express a fusion–fission process (**T**={*T*_*j*_}; **w**={*w*_*j*_}). *q* is the basal strength of the chemical fluxes. *τ*_*j*_ is the time at which the *j*-th fusion starts.

In general, the chemical reaction dynamics in a droplet open-reactor system (Fig. 1) is described as


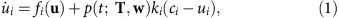


where *t* is time; *u*_*i*_ and *c*_*i*_ (*i*=1, 2,⋯) are the concentrations of chemicals *U*_*i*_ in a reactor droplet (reactor) and transporter droplets (transporters), respectively; *k*_*i*_ is the exchange rate of *U*_*i*_ caused by its diffusion during a fusion state; *f*_*i*_(**u**) (**u**={*u*_*i*_}) expresses chemical reactions; *p*(*t*; **T**, **w**) expresses a fusion–fission process as a square pulse-train function with two discrete values, that is, 0 (non-fusion state) or 1 (fusion state) (Fig. 1b); **T**={*T*_*j*_} and **w**={*w*_*j*_} (*w*_*j*_<*T*_*j*_); and *T*_*j*_ and *w*_*j*_ are the interval and duration of *j*-th fusion–fission event, respectively.

**Figure 2:**
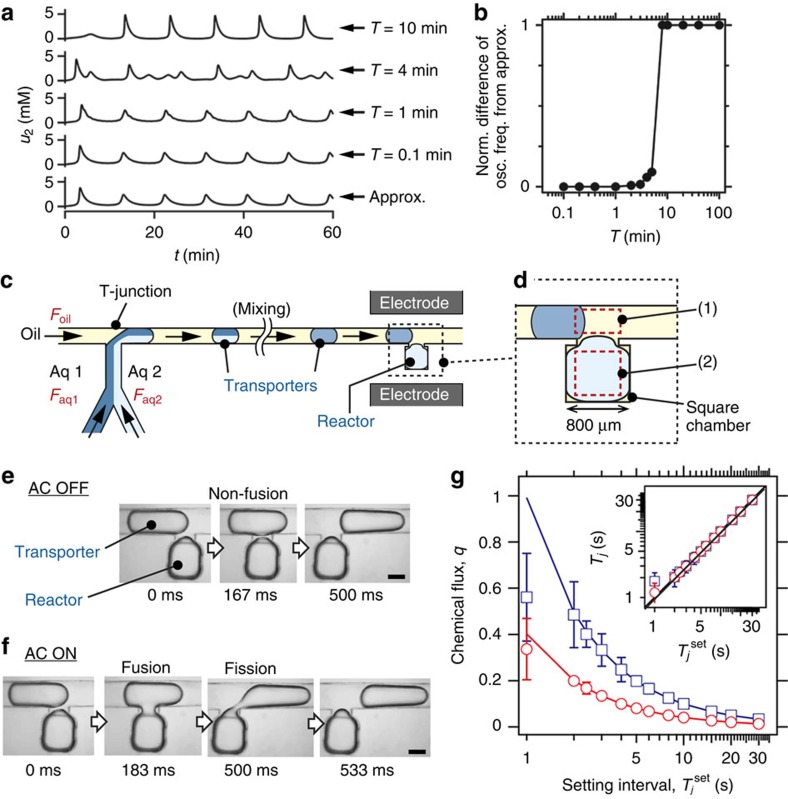
Characterization of the droplet open-reactor system. (**a**) Results of numerical simulations of the autocatalytic reaction shown by equation (2) in the droplet open-reactor system. The simulations were performed using the general form indicated by equation (1) for *T*=0.1–10 min, and using the approximate form described in equation (6) for ‘approx.' (details are given in the Methods section). *T*_*j*_=*T* and *w*_*j*_=*w* for all *j*. *w*/*T*=0.25 (fixed), which results in oscillation. (**b**) Normalized (Norm.) difference of the oscillation (osc.) frequency (freq.) from ‘approx.' in **a**. The value 0 indicates no frequency shift, whereas 1 means that the oscillation frequency is equivalent to the frequency of the fusion 1/*T*. Details are given in the Methods section. The solid line is provided as a guide for the eyes. (**c**) Design overview of the microfluidic system (details are given in Supplementary Fig. 2). Channel height, 500 μm. *F*_oil_, flow rate of the oil phase; *F*_aq1_ and *F*_aq2_, flow rate of the aqueous phases 1 and 2. (**d**) Enlarged view of the transporter and the reactor. The boxes outlined in red dashed lines indicate the areas in which (1) the detection of the transporter and (2) the fluorescence observation of the reactor were performed. (**e**,**f**) Bright-field microscope images of a fusion and fission event of a transporter and the reactor without and with a.c. voltage, respectively. Scale bars, 500 μm. (**g**) Control of *q* by *T*_*j*_^set^. Inset: control of *T*_*j*_ by *T*_*j*_^set^. The blue, red and black solid lines represent ideal values. Error bars: s.d. Sample size: 30 measurements. *F*_oil_=30 μl min^−1^ for **e** and **f**. *F*_oil_=15 μl min^−1^ (blue open square, *w*=0.99 s) and 30 μl min^−1^ (red open circle, *w*=0.40 s) for **g**. Solutions in the transporter and reactor were water for **e** and **f**, and 0.1-mM Fl–Na for **g**.

First, a reaction dynamics in a droplet open-reactor system is investigated using a simple two-variable (*u*_1_ and *u*_2_) autocatalytic reaction:


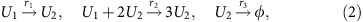


where *r*_1_, *r*_2_ and *r*_3_ are reaction rates, and *ϕ* indicates the degradation of *U*_2_. For this reaction system, we have *f*_1_(*u*_1_, *u*_2_)=−*r*_1_*u*_1_–*r*_2_*u*_1_*u*_2_^2^ and *f*_2_(*u*_1_, *u*_2_)=*r*_1_*u*_1_+*r*_2_*u*_1_*u*_2_^2^−*r*_3_*u*_2_. This type of reaction is known to require well-controlled sustained chemical fluxes and is widely observed in systems ranging from physicochemical to cellular^26^,^28^. The time courses of *u*_2_ for various fusion–fission periods are shown in Fig. 2a and Supplementary Fig. 1a (*T*=0.1–10 min), where the fusion–fission events are assumed to be periodic (that is, *T*_*j*_=*T* and *w*_*j*_=*w* for all *j*). When *T* is relatively small (*T*=0.1 and 1 min), the reaction exhibits its intrinsic behaviours: the limit cycle oscillation (Fig. 2a) and the convergence to a steady state (Supplementary Fig. 1a). In contrast, when *T* is relatively large (*T*≥4 min), the reaction appears to be disturbed by the droplet fusion and fission. Thus, an appropriate range of fusion–fission periods is required to achieve chemical reactions in the droplet open-reactor system.

Next, we mathematically analyse the fusion–fission process. A single fusion–fission event is expressed by *H*(*t*−*τ*_*j*_)−*H*(*t*−*τ*_*j*_−*w*_*j*_), where *τ*_*j*_ is the time at which the *j*-th fusion starts (that is, *τ*_*j*+1_−*τ*_*j*_=*T*_*j*_; Fig. 1b) and *H*(*t*) is a step function: *H*(*t*)=0 (*t*<0) or 1 (*t*≥0). Thus,


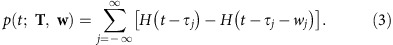


In the simplest case, namely, in which the fusion–fission events are periodic (*T*_*j*_=*T* and *w*_*j*_=*w* for all *j*), we have


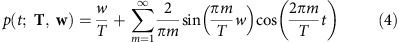


(details are given in Supplementary Note 1). Here *τ*_mac_ is defined as a characteristic time for the macroscopic dynamics of chemical reactions (for example, in the case of Fig. 2a, *τ*_mac_∼10 min). When 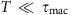, the second term in the right-hand side of the equation does not affect the reaction dynamics; thus,


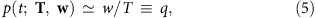


where *q* is a dimensionless parameter expressing the ratio of fusion states in the fusion–fission process (Fig. 1b). Therefore, the reaction dynamics in the droplet open-reactor system can be described by the following approximate form:


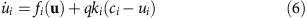


The reaction time course calculated using the approximate form (equation (6)) is shown in Fig. 2a (approx.) and is almost equivalent to the time courses of *T*=0.1 and 1 min shown in Fig. 2a. Figure 2b shows the difference between the time courses calculated by equations (1) and (6). These results indicate that the approximate form described by equation (6) can predict the reaction dynamics when 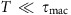 (∼10 min). Supplementary Fig. 1a (approx.) and 1b show the same result.

In summary, the reaction dynamics in the droplet open-reactor system is essentially regulated by *q*, which is the pulse density of the pulse-train function *p*. This type of control mechanism is called pulse-density modulation and is widely used in electrical devices and information–communication technologies because of its usefulness for parameter control.

### Construction of a droplet open-reactor system

Figure 2c,d shows the design overview of a droplet-based microfluidic system constituting the droplet open-reactor system (design details are given in Supplementary Fig. 2). The reactor used for chemical reactions consisted of a W/O microdroplet that was fixed in a square chamber in a microchannel between a pair of electrodes. The transporters were W/O microdroplets flowing through the microchannel. The diameter of the reactor was ∼800 μm, and its reaction volume was ∼0.3 μl. The diameter of the transporters was ∼500 μm, and the length along the flow was on the order of several hundred to one thousand micrometres, depending on the flow rate. The transporters were generated at the T-junction from two aqueous solutions (Supplementary Fig. 3a) and then delivered to the reactor after the solutions in the transporters were mixed through a zigzag channel^29^ (Supplementary Figs 2a and 3b). Fusion of the reactor and transporters was controlled by applying an a.c. voltage between the electrodes^30^,^31^,^32^. The reactor remained in a non-fusion state in the absence of the a.c. voltage (Fig. 2e and Supplementary Movie 1) and fused with the transporters on the application of the a.c. voltage (Fig. 2f and Supplementary Movie 2). The fused droplets were immediately fissioned by the shear stress of the oil flow and returned to their non-fusion states^30^. The fusion–fission event was then repeated. Because the volume of the reactor was limited to the square chamber and the reactor was stabilized by its own surface tension, its reaction volume was kept almost constant.

In this microfluidic device, a droplet-fusion control programme was used to precisely control the fusion–fission interval *T*_*j*_ according to a set value 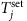 (Supplementary Fig. 4). The droplet-fusion control programme monitored the positions of the transporters and the fluorescence intensity of the reactor (Fig. 2d). On the basis of the monitoring information, the droplet-fusion control programme controlled the fusion and fission by switching the a.c. voltage on/off.

Using this system, we investigated the controllability of the fusion–fission process. The duration of the fusion state *w* was determined according to the oil flow rate *F*_oil_ (Supplementary Fig. 3c). Figure 2g shows the relationship between *q* and *T*_*j*_^set^, and its inset shows the relationship between *T*_*j*_ and *T*_*j*_^set^. When *T*_*j*_^set^≥2 s, *T*_*j*_=*T*_*j*_^set^, and thus, *T*_*j*_ was successfully controlled. However, when *T*_*j*_^set^<2 s, *T*_*j*_>*T*_*j*_^set^ (inset) because the arrival interval of the transporters was comparable to *T*_*j*_^set^. As a result *q* was well controlled when *T*_*j*_^set^≥2 s. Supplementary Fig. 3d shows that the inner solution of the reactor (initially pure water) was completely exchanged with a fluorescein sodium (Fl–Na) solution in the transporters after several tens of fusion events with the transporters, indicating that ∼1–2% of the chemicals were exchanged in each fusion–fission event. The inner solution of the reactor after a single fusion was homogenized within ∼60 s, much faster than homogenization through the simple diffusion of molecules (∼600 s). This faster mixing resulted from a rotating flow in the reactor induced by the oil flow in the microchannel (Supplementary Fig. 3e)^30^.

### Control over chemical reaction dynamics far from equilibrium

We investigated the controllability of chemical reaction dynamics far from equilibrium using a droplet open-reactor system through bromate–sulfite–ferrocyanide (BSF) pH oscillation^33^,^34^,^35^ (details are given in the Methods section, Supplementary Note 2 and Supplementary Table 1). This reaction consists of a time-delay negative feedback loop of the autocatalytic production of H^+^ and the consumption of H^+^ at low pH. This reaction requires BrO_3_^−^, Fe(CN)_6_^4−^ and SO_3_^2−^ as substrates, and generates a limit cycle oscillation of pH only when appropriate chemical fluxes are maintained. In addition to the fact that the reaction mechanism has been well documented, the strict requirement for appropriate chemical fluxes is suitable for investigating the performance of an open reactor unlike other nonlinear chemical reactions, such as the Belousov–Zhabotinsky reaction^26^, which exhibit relatively stable transient chemical oscillations even in chemically closed conditions.

**Figure 3:**
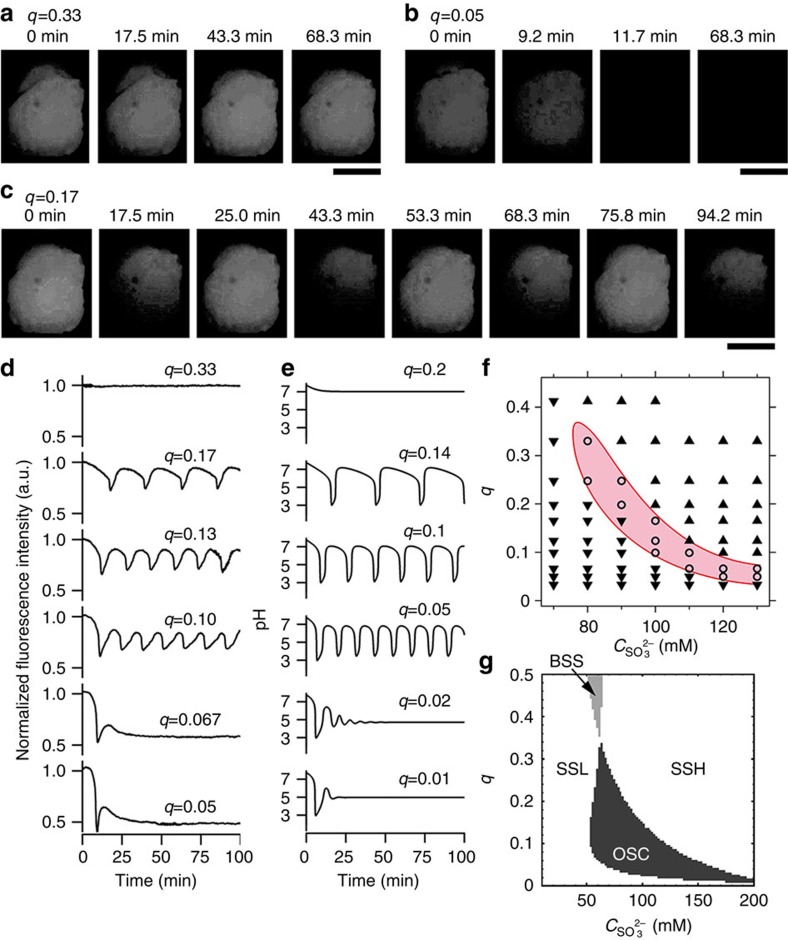
Control of chemical oscillation dynamics using the droplet open-reactor system. (**a**–**c**) Fluorescence microscope images of the reactor. High intensity (white) indicates high pH, and vice versa (Supplementary Fig. 5). Scale bars, 500 μm. (**a**) Convergence to a steady state at a higher pH (SSH). (**b**) Convergence to a steady state at a lower pH (SSL). (**c**) Limit cycle oscillation of pH (OSC). (**d**) Time courses of fluorescence intensity in the reactor. (**e**) Numerical simulation of **d**. (**f**) 2D bifurcation diagram. Open circle: limit cycle oscillation; filled triangle: SSH; filled inverted triangle: SSL. (**g**) 2D bifurcation diagram calculated using linear stability analysis. White: monostable steady state of SSH or SSL; light grey: bistable steady state (BSS) of SSH and SSL; dark grey: OSC. 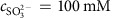 for **a**–**e**. The compositions of the solutions in the transporter and reactor are given in the Methods section.

In the experiments all the substrates were supplied to the reactor using transporters. The pH change in the reactor was observed via the fluorescence intensity change of a pH indicator (Fl–Na; Fig. 3a–c). The value of *q* was varied by changing *T*_*j*_ (*w* was fixed at 0.99 here and in further experiments). When *q* was high (*q*=0.33), the reaction converged to a steady state at a higher pH (SSH; higher intensity) (Fig. 3a and Supplementary Movie 3). When *q* was low (*q*=0.05), the reaction converged to a steady state at a lower pH (SSL; Fig. 3b and Supplementary Movie 4). Under the intermediate condition (*q*=0.17), the intensity in the reactor exhibited pH oscillation (Fig. 3c and Supplementary Movie 5). Spatial heterogeneity was observed in these results, possibly because of the non-instantaneous mixing of the inner solution of the reactor (Supplementary Fig. 3e, Supplementary Note 3 and Supplementary Fig. 6)^36^. Figure 3d shows the time courses of the fluorescence intensity; bifurcation among SSH, pH oscillation and SSL was observed by changing *q* as a bifurcation parameter. The observed time courses are in semi-quantitative agreement with the numerical simulation results (Fig. 3e, details are given in Supplementary Note 2). Figure 3f shows a two-dimensional (2D) bifurcation diagram when *q* and 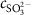 were used as bifurcation parameters (all of the time-course data are shown in Supplementary Fig. 7). The result agrees with the 2D bifurcation diagram calculated using linear stability analysis (Fig. 3g, details are given in Supplementary Note 2). The bistable steady state shown in Fig. 3g was not experimentally observed because it was difficult to precisely control *q* within the bistable steady-state region (Fig. 2g, blue open squares; *T*_*j*_^set^≤2 s). In addition, we observed the similar reaction behaviours in arrayed multiple reactors (Supplementary Fig. 8). In summary, the results show that the chemical dynamics far from equilibrium was successfully controlled using the droplet open-reactor system.

### Time-variable external control of chemical reaction dynamics

**Figure 4:**
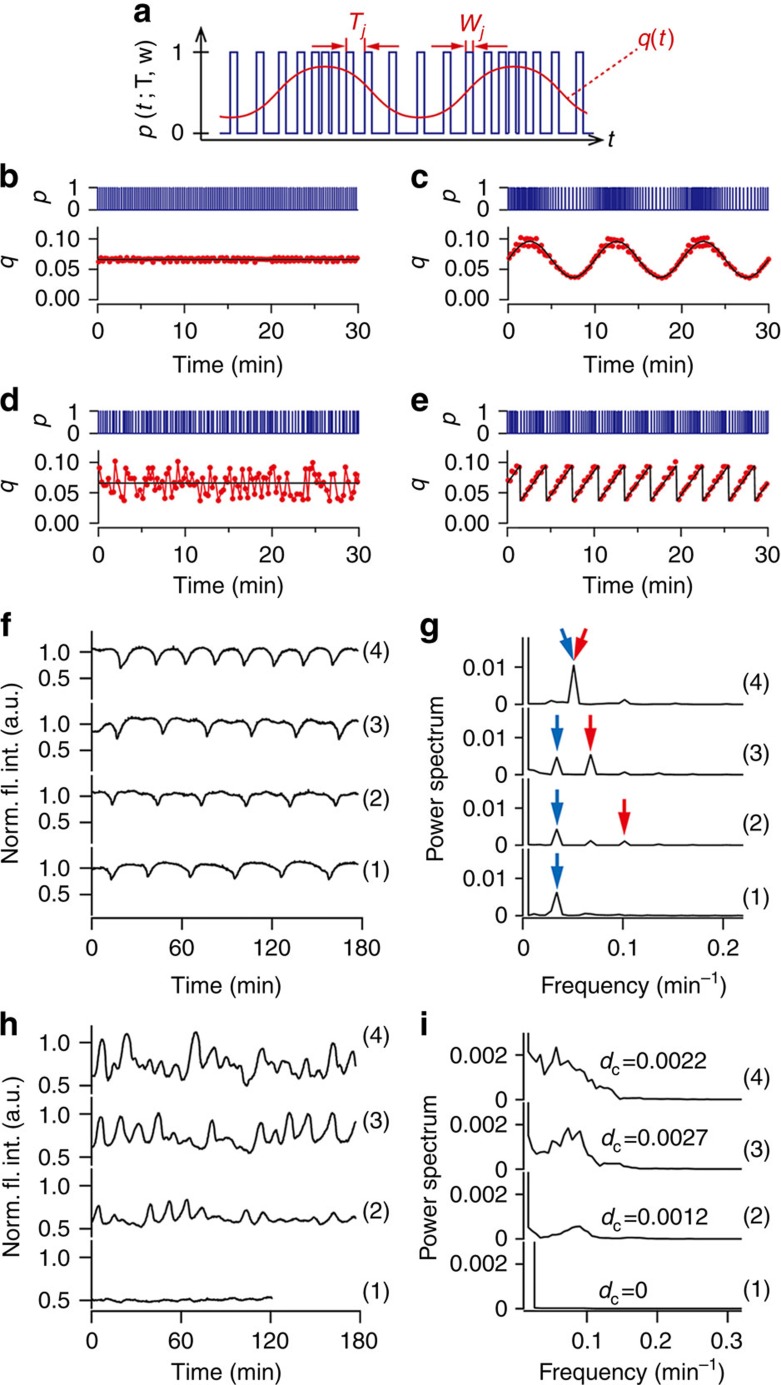
Time-variable external control of chemical oscillation dynamics using the droplet open-reactor system. (**a**) Schematic diagram of the pulse-density modulation control of time-variable *q*(*t*). When the pulse train of *p*(*t*; **T**, **w**) is denser, *q*(*t*) is higher, and vice versa. (**b**–**e**) Generation of *p* and *q*. Blue lines: pulse trains of *p*(*t*; **T**, **w**); black lines: (**b**,**c**,**e**) theoretical curves of *q*(*t*) and (**d**) theoretical average of *q*(*t*); red dots and lines: *q*(*t*) generated in experiments, calculated as *q*(*t*=*τ*_*j*_)=*w*_*j*_/*T*_*j*_. *w*_*j*_=0.99 s (fixed for all *j*). (**b**) Constant (*Z*_C_(*t*)). (**c**) Sinusoidal wave (*Z*_S_(*t*), *A*_*q*_=0.45, *T*_*q*_=10 min). (**d**) White noise (*Z*_N_(*t*), *A*_*q*_=0.45). (**e**) Saw-tooth wave functions (*Z*_St_(*t*), *A*_*q*_=0.45, *T*_*q*_=3 min). *Z*_C_(*t*), *Z*_S_(*t*), *Z*_N_(*t*) and *Z*_St_(*t*) are described in detail in the Methods section. The baseline value of *q*(*t*): 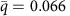. (**f**) Entrainment of the chemical oscillation to the external sinusoidal signal. 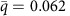. (1) *A*_*q*_=0 (without sinusoidal signal) and (2–4) *A*_*q*_=0.45. (2) *T*_*q*_=10 min, (3) *T*_*q*_=15 min and (4) *T*_*q*_=20 min. (**g**) Power spectra of **f**. (**h**) Noise-induced pulsed excitation when white noise was added to SSL. 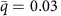. (1) *A*_*q*_=0 (without noise), (2) *A*_*q*_=0.15, (3) *A*_*q*_=0.3 and (4) *A*_*q*_=0.45. (**i**) Power spectra of **h**. 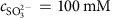. The compositions of the solutions in the transporter and reactor are given in the Methods section. Norm. fl. int., normalized fluorescence intensity.

Here we extended equation (5) to the case in which *T*_*j*_ and *w*_*j*_ are variable for each fusion–fission event *j*:


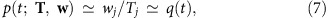


where *q*(*t*) is a time-variable function (Fig. 4a). Figure 4b–e shows various waveforms of the chemical fluxes *q*(*t*) that were produced through pulse-density modulation (details are given in the Methods section). When the pulse train of *p* had equally spaced intervals, *q* was constant (Fig. 4b). Figure 4c shows the resulting sinusoidal waveform. When *T*_*j*_^set^ was randomly generated so that *q*(*t*) followed a uniform distribution, *q* exhibited white noise (Fig. 4d). Figure 4e shows a saw-tooth wave with a 3-min period that demonstrates sharp switching of chemical fluxes. Similarly, different waveforms including square waves were produced (Supplementary Note 4 and Supplementary Fig. 9).

We demonstrated external control of chemical reactions using time-variable chemical fluxes. First, Fig. 4f,g shows the BSF pH oscillation time courses and their Fourier power spectra obtained when the chemical fluxes included a sinusoidal external signal. When the periods of the added sinusoidal signal and the chemical oscillation were substantially different, additional periods appeared in the chemical oscillation ((2) and (3) in Fig. 4f,g; red arrows). In contrast, when they were similar, the period of the intrinsic chemical oscillation was entrained to that of the added sinusoidal signal ((4) in Fig. 4f,g; blue and red arrows). Next, we investigated the effect of the noise on the chemical reactions in SSL by changing the noise strength included in the chemical fluxes (Fig. 4h,i). When the noise was weak, noise-induced pulsed excitations randomly occurred ((2) in Fig. 4h,i). However, when the noise was relatively strong, the excitation timing was more coherent ((3) and (4) in Fig. 4h,i). We calculated then the degree of coherence, *d*_c_ (definition is given in the Methods section)^37^,^38^. *d*_c_ has a maximum at (3) in Fig. 4i, which suggests that noise was too strong and disturbed the pulsed excitations ((4) in Fig. 4i). This coherent phenomenon is called coherence resonance^37^,^38^. Thus, based on these results, complex nonlinear chemical phenomena observed far from equilibrium can be quantitatively studied using the droplet open-reactor system.

### Autonomous feedback control of chemical reaction dynamics

**Figure 5:**
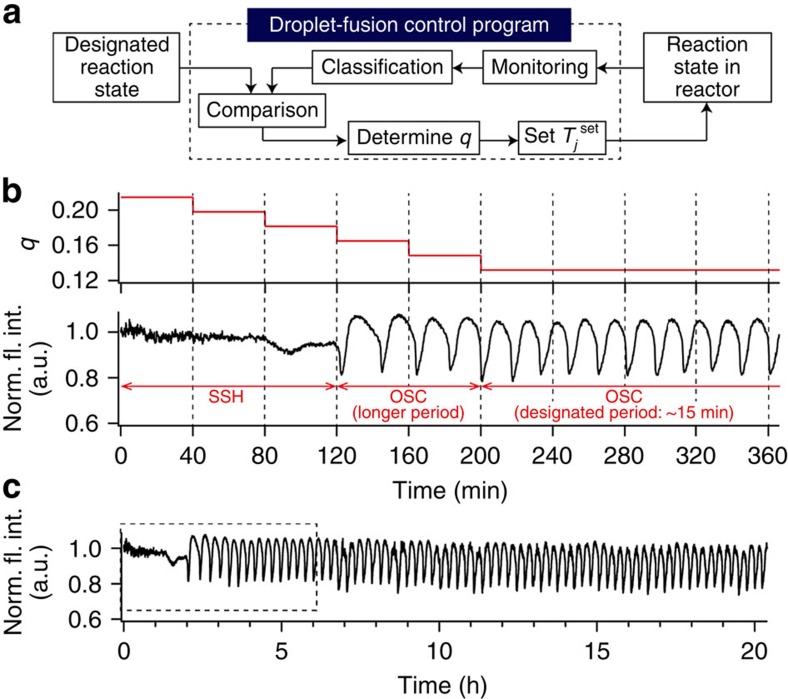
Feedback control of BSF pH oscillation using the droplet open-reactor system. (**a**) Scheme of feedback control. The droplet-fusion control programme fluorescently monitors and classifies the current reaction state in the reactor (SSH, SSL and oscillation of pH). The droplet-fusion control programme compares the reaction state with a designated one; when they are different, the droplet-fusion control programme automatically changes *q* by changing *T*_*j*_^set^ to obtain a reaction state closer to the designated one. (**b**) Time course of *q* (upper) and normalized fluorescence intensity (Norm. fl. int.; lower) during feedback control (designated period: 15 min). Initial condition *q*=0.215 (*T*_*j*_=4.62 s). The change in *q* per step was 0.0165. (**c**) Long-term observation of oscillation after applying the feedback control in **b** (box indicated by dashed lines). The compositions of the solutions in the transporter and reactor are given in the Methods section.

Finally, we investigated autonomous feedback control of chemical reaction dynamics far from equilibrium in the droplet open-reactor system, by extending the control method used for time-variable chemical fluxes. The feedback scheme is shown in Fig. 5a (the detailed algorithm is given in Supplementary Figs 10 and 11). Figure 5b shows the result of feedback control when the designated reaction state was set to ‘oscillation with a period of 15 min'. The experiments started from SSH. The droplet-fusion control programme changed *q* every 40 min (Fig. 5b, upper graph) in response to the monitored reaction state. As a result, the chemical reaction system reached the designated state at ∼200 min (Fig. 5b, lower graph). Figure 5c shows a long-term (>20 h) observation of the sustained pH limit cycle oscillation after applying the feedback control in Fig. 5b. This observation indicates that the droplet open-reactor system can stably ‘incubate' the controlled dynamical chemical reactions far from equilibrium over the long term.

## Discussion

In this study, we developed a droplet open-reactor system that can finely and dynamically control chemical fluxes. Its control mechanism was mathematically formulated as pulse-density modulation control and was implemented using electrical control of the fusion and fission of droplets. Using the pulse-density modulation control, we produced various waveforms such as sinusoidal waves, saw-tooth waves and white noise. We first demonstrated control over a dynamical chemical reaction system far from equilibrium. The current system exhibited spatial heterogeneity of the chemical reaction (Fig. 3c and Supplementary Note 3) resulting from the non-instantaneous mixing of the inner solution because of the reactor size (several hundred micrometres in diameter). However, this issue will not occur in a smaller microreactor as we previously reported^30^. In addition, in some situations, the spatial heterogeneity may be utilized to investigate dynamically changing spatial patterns of chemical concentrations. In addition, the accurate chemical concentrations in the reactor could not be determined because of the volume measurement error (∼9%) associated with the bright-field microscope images. However, the experimental error in the chemical concentration control is thought to be relatively small, as is suggested by the experiment illustrated in Fig. 5c, which shows the stable long-term limit cycle oscillation of pH. For highly quantitative analyses of reactions, especially in the case of using a smaller reactor droplet, a more accurate method for volume measurements, such as the use of a high-speed confocal microscope, will be required. Next, we performed external control of the reaction system, which has not been achieved previously at the microscale even though it is essential for the study of nonlinear chemical reactions and well studied in beaker-sized open reactors^39^. Finally, we demonstrated autonomous feedback control over the reaction system. In the feedback experiments, we used optical read-outs to monitor the reaction states, but alternative read-outs, such as electrical measurements, could be used in this system by introducing micropatterned electrodes. Combining multiple measurements to determine the reaction state will expand the abilities of this system.

To date, several microchannel-based open reactors have been reported^20^,^21^,^22^. Similar to these microfluidic open reactors, our droplet open-reactor system has the advantages of low sample consumption compared with conventional beaker-sized open reactors, facilitating microscale bio-inspired engineering. In the current setup, all chemicals flow into/out of the reactor, and selective chemical fluxes cannot be achieved, unlike on-chip DNA compartment reactors^22^. However, this capability will be improved by the immobilization of chemicals on solid surfaces, such as microbeads. In addition, the droplet open-reactor system has an advantage over previous open microreactors in terms of the controllability of chemical fluxes. The better controllability of this system is attributable to two-phase-flow microfluidics, in which the reaction solutions compartmentalized in droplets and the transporting fluids can be separately manipulated^40^,^41^. As a result, the addition/removal of chemicals can be achieved through pulse-density modulation control of the frequency of the digitalized droplet fusions that are electrically switched. Thus, manipulation of the whole solution in the channels and tubes is not required, and complicated microfluidic components, such as valves and mixers, are unnecessary. These characteristics allow open reactors to be combined with sophisticated control methods, such as the electrical^42^,^43^,^44^ and optical^45^ manipulation of fluids. In addition, because the on/off switching response was rapid compared with that of traditional beaker-sized open reactors and other microfluidic open reactors^20^,^21^, sharp waveforms, such as saw-tooth and white noise waves, were also achieved. As a result, we successfully investigated the coherence resonance, which is usually difficult to identify because the intentionally produced noise must be properly controlled to prevent it from being buried in the intrinsic noise of the experimental system. This control was achieved by the precise and rapid control of chemical fluxes. However, based on the mathematical and experimental results, we identified a few limitations caused by the use of droplets as transporters. First, the controllability of *q* decreases at high values of *q* (Fig. 2g) because the fusion interval (*T*_*j*_) cannot be less than the arrival time of the transporters. In addition, *T*_*j*_ values close to or exceeding the characteristic time of the macroscopic reaction dynamics cannot be used (Fig. 2b and Supplementary Fig. 1b) because the discreteness of the fusion–fission process affects the chemical reaction dynamics (that is, *q* that are too low cannot be used). In summary, there is an appropriate range of *q* necessary to control a chemical reaction dynamics in this system.

When this system is extended to coupled multiple reactors, it will be even more useful for the study of chemical reactions far from equilibrium. For example, multiple reactors linearly arrayed along a microchannel (for example, Supplementary Fig. 8a) could be diffusionally coupled with each other if semipermeable walls are constructed between the chambers. In addition, multiple reactors could contain different chemicals if the chemicals are injected through the top injection holes of the chambers (Supplementary Fig. 2) and then kept separate by fixation on solid surfaces, such as microbeads. Larger-scale coupled reactors arrayed in a 2D manner could be constructed and accessed through three-dimensional microchannels. This type of open-reactor system will facilitate studying the dynamical population behaviours of chemical reactions far from equilibrium^13^.

We believe that the droplet open-reactor system can be applied to many complex reaction systems such as artificial DNA circuits and gene circuits^46^,^47^,^48^,^49^,^50^, metabolic systems^51^,^52^ and microchemostat-like reactors^53^, in synthetic biology. In addition, the precise dynamical control of non-equilibrium chemical reactions will promote the rational design of oscillating enzymatic networks^54^ and the production of complex and hierarchical materials, including biomineralization far from equilibrium^3^. In particular, our method has good compatibility with system control theory and computational intelligence because of the controllability achieved using pulse-density modulation. In the future, this method may therefore be applied to system control biology^55^ based on the real-time monitoring and model-driven control of living cells or artificial cell-like systems^56^,^57^,^58^,^59^,^60^,^61^ based on software–wetware hybrids.

## Methods

### Numerical simulations of the two-variable model

The parameter values used in the numerical simulation of equation (2) are as follows: *r*_1_=0.04 min^−1^, *r*_2_=1 mM^−2^ min^−1^, *r*_3_=1 min^−1^, *k*_1_=*k*_2_=0.4 min^−1^, *c*_1_=10 mM and *c*_2_=0 mM. The vertical axis in Fig. 2b indicates the degree of the oscillation-frequency shift, calculated using the maximum peak of the fast Fourier transformation spectra: (*f*_*T*, max_−*f*_approx., max_)/(*f*_fusion_−*f*_approx., max_), where *f*_fusion_=1/*T*.

### Fabrication of the microfluidic device

The fabrication details are given in Supplementary Note 5 and Supplementary Fig. 2. The microfluidic system was constructed using two poly(methyl methacrylate) plates (1 mm thickness) because poly(methyl methacrylate) microchannels are stable for long periods without liquid swelling and are amenable to repeated use. The microchannel was fabricated on the upper plate using a fine-milling machine (MDX-40A, Roland DG). The upper and lower plates were attached by thermal compression bonding. At the beginning of the experiments, a reactor was introduced into the square chamber through a top injection hole using a micropipette; the hole was then sealed with transparent cellophane tape. The oil phase and aqueous phases were then flowed using microsyringe pumps (LEGATO180, KD Scientific) and disposable syringes (10 ml, SS-10SZ, Terumo). The electrodes were connected to a function generator (WF1974, NF Corporation) through a voltage amplifier (M-2629B-2CH, MESS-TEK). The droplet-fusion control programme was developed using an image-processing module of the OpenCV library and Microsoft Visual C++ (Microsoft Corporation).

### General experimental conditions

The oil phase consisted of mineral oil (Nacalai Tesque) containing 0.5% Span80 (Tokyo Chemical). Aqueous phases 1 and 2 were water for Fig. 2e,f. For Figs 2g and 4b–e, aqueous phase 1 was 0.2-mM Fl–Na (Sigma-Aldrich) and aqueous phase 2 was water. The aqueous phases used in the other cases are described in the Methods section reporting the BSF pH oscillation. *F*_aq1_=*F*_aq2_=10 μl min^−1^ for all experiments. *F*_oil_=15 μl min^−1^ (that is, *w*=0.99 s) for Figs 3, 4, 5. *F*_oil_ was varied for each experiment in Fig. 2. The a.c. voltage applied to effect droplet fusion was *V*_pp_=300 V (peak to peak; 1 kHz).

All the experiments were carried out using a fluorescence microscope (IX71 or IX81, Olympus). For the observations in Fig. 2e,f, a high-speed complementary metal-oxide-semiconductor camera (FASTCAM SA3 120K, Photron) was used. For the experiments in Figs 2g, 3, 4, 5, a digital camera (EOS 60D, Canon) was used.

The reproducibility of all experimental results was confirmed by performing at least two or three experiments.

### Experiments and numerical simulations of BSF pH oscillation

The following is a simple reaction model of the BSF reaction that is called the Rabai–Kaminaga–Hanazaki model^34^,^35^:


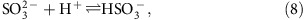



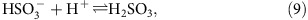



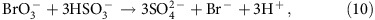



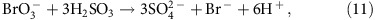



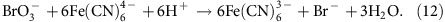


For Figs 3, 4f,h and 5, aqueous phase 1 consisted of 150-mM KBrO_3_ (Wako Pure Chemical) and 0.2-mM Fl–Na, whereas aqueous phase 2 was 30-mM K_4_Fe(CN)_6_ (Wako Pure Chemical), 15-mM H_2_SO_4_ (Nacalai Tesque) and Na_2_SO_3_ (Wako Pure Chemical). Thus, the final concentrations in the transporters were 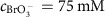, 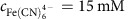, 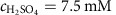 and 0.1-mM Fl–Na. 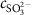 was varied depending on the experiment being performed. The initial chemical concentrations in the reactor were the same as those in the transporters. All the experiments were carried out at ∼35 °C using a thermo-plate (MATS-U52RA26, Tokai Hit) on the microscope.

We used the Rabai–Kaminaga–Hanazaki model for the numerical analyses in Fig. 3e,g. The numerical analyses were performed using Mathematica (Wolfram Research). The details are given in Supplementary Note 2.

### Time-variable control over chemical fluxes by pulse-density modulation

To generate time-variable chemical fluxes, we used 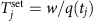 and 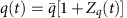, where *t*_0_=0; 
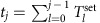
; and 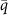 is the baseline value of *q*(*t*). *Z*_*q*_(*t*) was varied according to designated functions as follows: *Z*_C_(*t*)=0 for constant functions; *Z*_S_(*t*)=*A*_*q*_sin(2*πt*/*T*_*q*_) for sinusoidal wave functions; *Z*_N_(*t*)=*A*_*q*_*U*(−1, 1) for white noise functions, where *U*(−1, 1) indicates uniform random numbers between [−1, 1]; and *Z*_St_(*t*)=*A*_*q*_[2*R*(*t*, *T*_*q*_)/*T*_*q*_−1] for saw-tooth wave functions, where *R*(*t*, *T*_*q*_) gives the residue obtained when *t* is divided by *T*_*q*_. Details are given in Supplementary Note 4.

### Analysis of the noise-induced pulsed excitation

We produced the Fourier power spectra in Fig. 4i by five-point smoothing of the fast Fourier transformation results in Fig. 4h. From Fig. 4i, the degree of coherence^37^,^38^ was calculated by *d*_c_≡*h*(Δ*f*/*f*_p_)^−1^, where *f*_p_ is the peak frequency, and *h* and Δ*f* are the peak height and the peak width at half height, respectively.

## Additional information

**How to cite this article:** Sugiura, H. *et al*. Pulse-density modulation control of chemical oscillation far from equilibrium in a droplet open-reactor system. *Nat. Commun.* 7:10212 doi: 10.1038/ncomms10212 (2016).

## Supplementary Material

Supplementary Information Supplementary Figures 1-11, Supplementary Table 1, Supplementary Notes 1-5 and Supplementary References

Supplementary Movie 1 The movie data for Fig. 2e. The reproduction speed is 0.1-times speed.

Supplementary Movie 2 The movie data for Fig. 2f. The reproduction speed is 0.1-times speed.

Supplementary Movie 3 The movie data for Fig. 3a. The reproduction speed is 500-times speed.

Supplementary Movie 4 The movie data for Fig. 3b. The reproduction speed is 500-times speed.

Supplementary Movie 5 The movie data for Fig. 3c. The reproduction speed is 500-times speed.
