# Supplementary material for: Pulse-density modulation control of chemical oscillation far from equilibrium in a droplet open-reactor system
Source: Nat Commun. 2016 Jan 20;7:10212. doi: 10.1038/ncomms10212 (PMC4735724; doi:10.1038/ncomms10212)
Supplement: Supplementary Information — Supplementary Figures 1-11, Supplementary Table 1, Supplementary Notes 1-5 and Supplementary References [file ncomms10212-s1.pdf]

Supplementary Figures

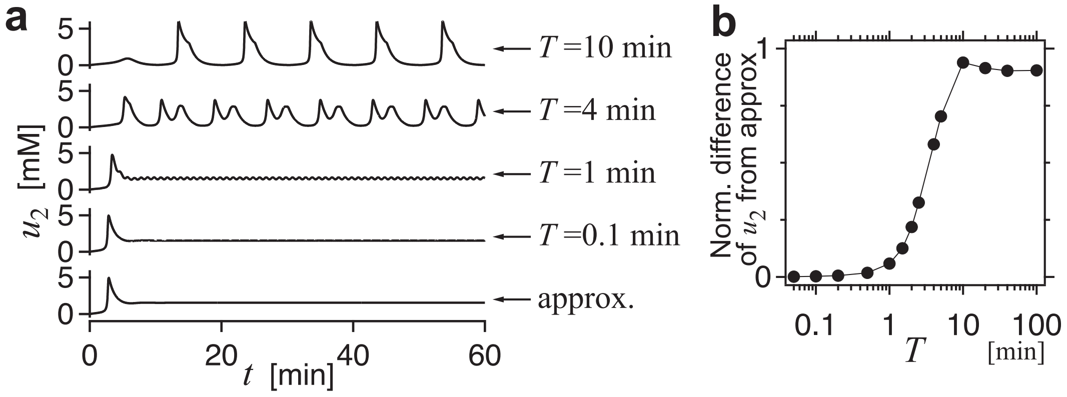

Supplementary Figure 1. Numerical characterisation of the droplet open-reactor system. (**a**) Numerical simulations of the autocatalytic reaction shown in Eq. 2 (in the main text) in the droplet open-reactor system. The simulations were performed using the general form shown in Eq. 1 (in the main text) for $T = 0.1$–10 min, and using the approximate form represented by Eq. 6 (in the main text) for "approx." (Details are given in the Methods section in the main text). $T_j = T$ and $w_j = w$ for all j. $w/T = 0.5$ (fixed), which results in convergence to a steady state. (**b**) Normalised difference of u_2 from "approx." in (**a**), which is calculated as the time-averages of the difference of u_2 normalised by dividing by the steady state values of u_2 (approx.); i.e. $\left\langle u_{2,T}(t) - u_{2,\text{approx.}}(t) \right\rangle_t / u_{2,\text{approx.}}(t = 60)$. A value of 1 means that the difference is ±100%, whereas a value of 0 indicates no difference. The solid line is provided as a guide for the eyes.

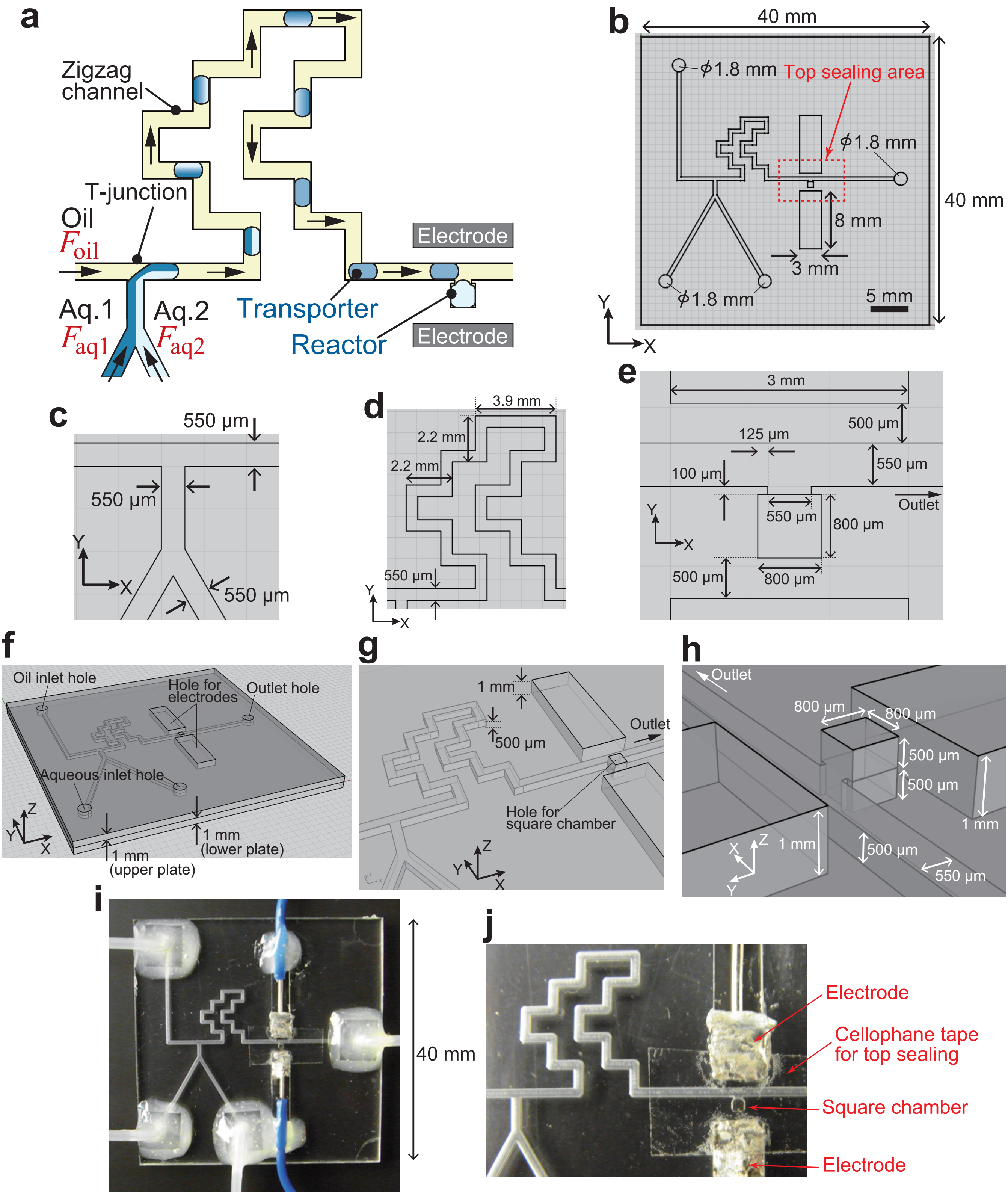

Supplementary Figure 2. Design details of microfluidic device for droplet open-reactor system. (**a**) Schematic diagram of microfluidic device. (**b**) Top view. (**c**) Enlarged top view of T-junction for transporter generation. (**d**) Enlarged top view of zig-zag channel for mixing. (**e**) Enlarged top view of square chamber for reactor. (**f**) and (**g**) 3D wide views. The microchannel was fabricated in the upper plate. The lower plate was attached to the upper plate by thermal compression bonding. (**h**) Enlarged 3D view of the square chamber for a reactor. The design and illustration were created using 3D CAD software (Rhinoceros 3D, Robert McNeel & Associates). (**i**) and (**j**) Photographs of fabricated microfluidic system.

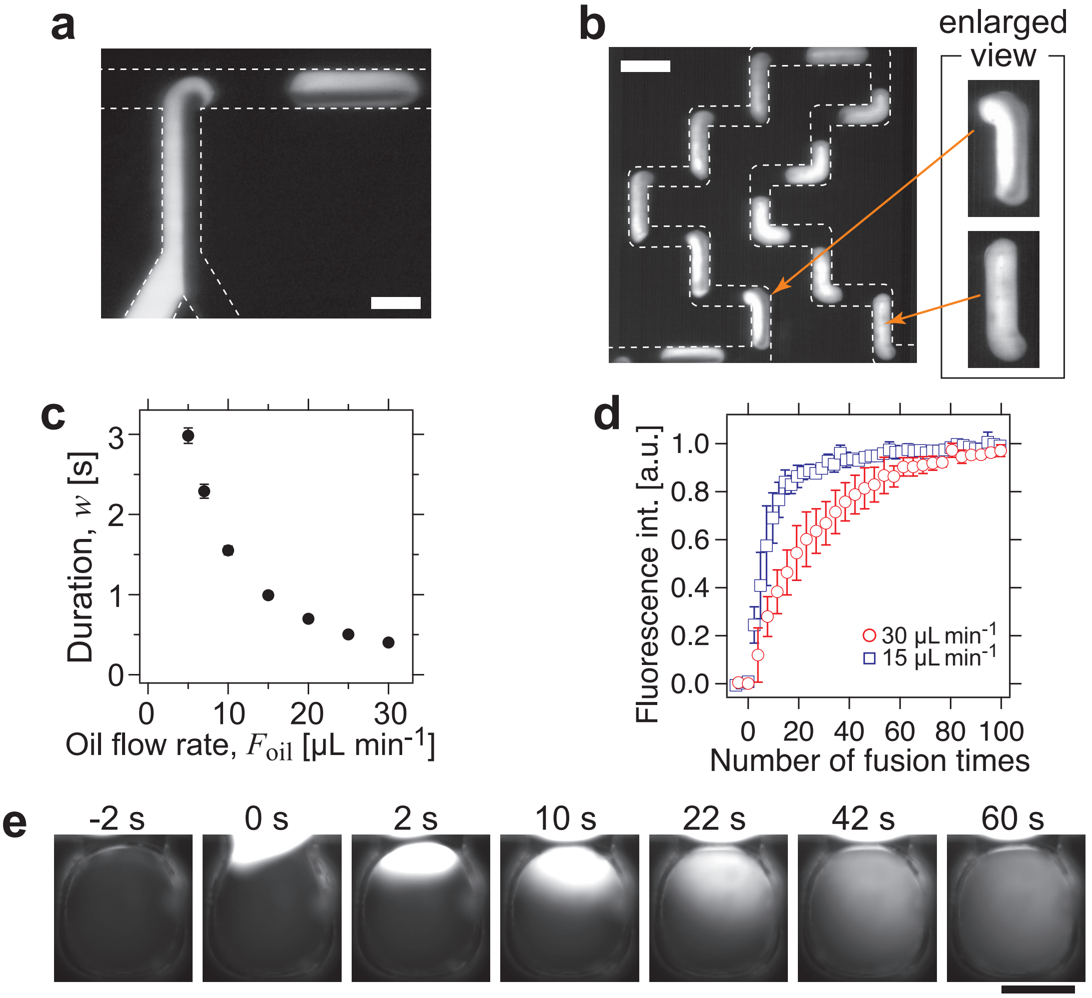

Supplementary Figure 3. Experimental characterisation of the droplet open-reactor system. (**a**) Generation of a transporter at the T-junction. Scale bar: 500 μm. (**b**) Solution mixing in the transporters in the zigzag mixing channel. Scale bar: 1 mm. (**c**) Control of w by F_{oil}. T_j^{set} = 8 s (fixed). (**d**) Solution exchange by successive multiple fusions. (**e**) Fluorescently observed diffusion of chemicals in the reactor immediately after a single fusion-fission event. The mixing time (~ 60 s) was much faster than that of the simple diffusion of molecules (~ 600 s) because of rotating flow in reactor [5]. Scale bar: 500 μm. Aqueous phase 1: 0.2 mM fluorescein sodium for (**a**)-(**c**) and 1 mM fluorescein sodium for (**d**) and (**e**). Aqueous phase 2: water for all. Oil phase: mineral oil with 0.5% Span80 for (**a**)–(**c**) and mineral oil with 5% Span80 for (**d**) and (**e**). F_{oil} = 20 μL min^{-1} for (**a**) and (**b**), and 15 μL min^{-1} for (**e**). F_{oil} = 15 μL min^{-1} (blue open square, w = 0.99 s), and 30 μL min^{-1} (red open circle, w = 0.40 s) for (**d**). F_{aq1} = F_{aq2} = 10 μL min^{-1} in all cases. (**a**) and (**b**) were captured using a high-speed CMOS camera (FASTCAM SA3 120K, Photron), (**c**) was captured using a digital camera (EOS 60D, Canon), and (**d**) and (**e**) was captured using a scientific CMOS camera (Zyla-5.5-CL3, Andor). Error bars of (**c**) and (**d**): s.d. Sample size of (**c**) and (**d**): 10 and 3 measurements, respectively.

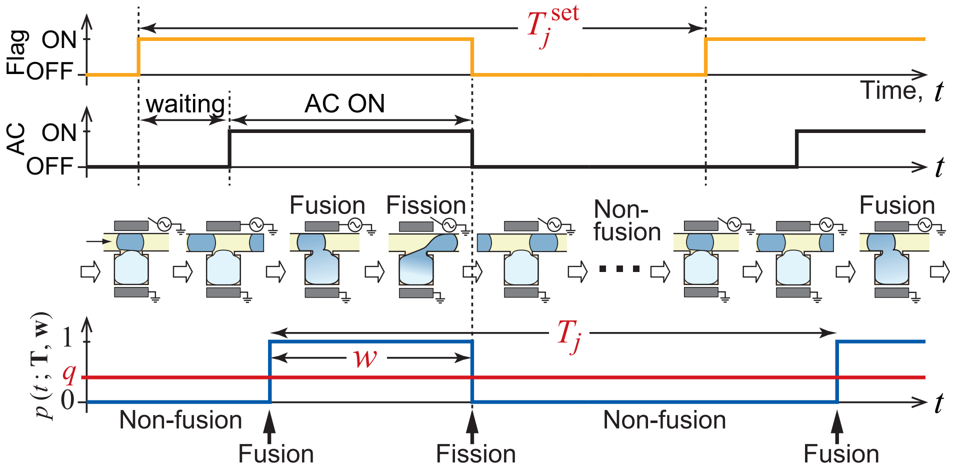

Supplementary Figure 4. Control mechanism of fusion and fission by a droplet-fusion control program. T_j^{set}: set value of j-th fusion-fission interval; T_j and w: actual interval and duration of j-th fusion-fission, respectively. $p(t; \mathbf{T}, \mathbf{w})$: pulse-train function expressing fusion-fission process. q: basal strength of chemical fluxes. First, a fusion flag in the droplet-fusion control program is turned ON by following T_j^{set}; the droplet-fusion control program then waits for passing of a transporter to prevent unintended fusion, and AC voltage is turned ON; when the next transporter comes, the transporter fuses with the reactor; finally, just after the fission of the transporter, the flag and AC voltage are turned OFF; this cycle is repeated.

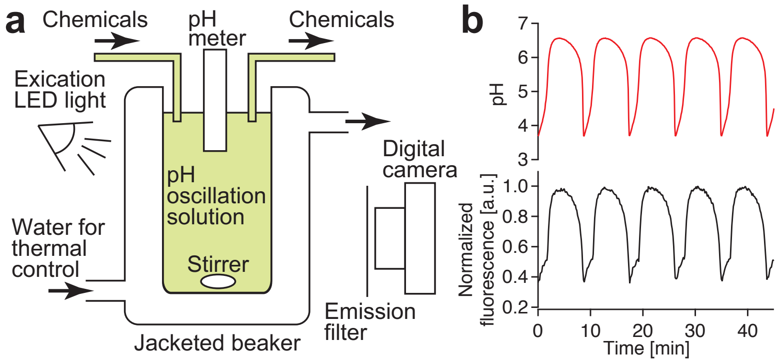

Supplementary Figure 5. Comparison of pH changes and fluorescence intensity changes of BSF pH oscillation in beaker-sized open flow-reactor. (**a**) Experimental setup. (**b**) Monitoring of BSF pH oscillation in beaker-sized open flow-reactor, based on fluorescence intensity (black line) and pH (red line). The experiments were carried out in a jacketed beaker (0065-01-13-01, Tokyo Glass Kikai). The total reaction volume was 18 mL, and 75 mM $KBrO_3$, 15 mM $K_4Fe(CN)_6$, 7.5 mM H_2SO_4, 100 mM Na_2SO_3, and 1 mM fluorescein sodium were flowed into the beaker at flow rates 0.9 mL min^{-1} using peristaltic pumps (MP-1000, EYELA). 40°C water was run into the beaker jacket to maintain the solution temperature. Fluorescence was observed using a digital camera (EX-F1, Casio). Excitation wavelength of light-emitting diode light: 470–475 nm. Emission filter wavelength > 540 nm. The pH was monitored using a pH meter (D-52LAB, Horiba).

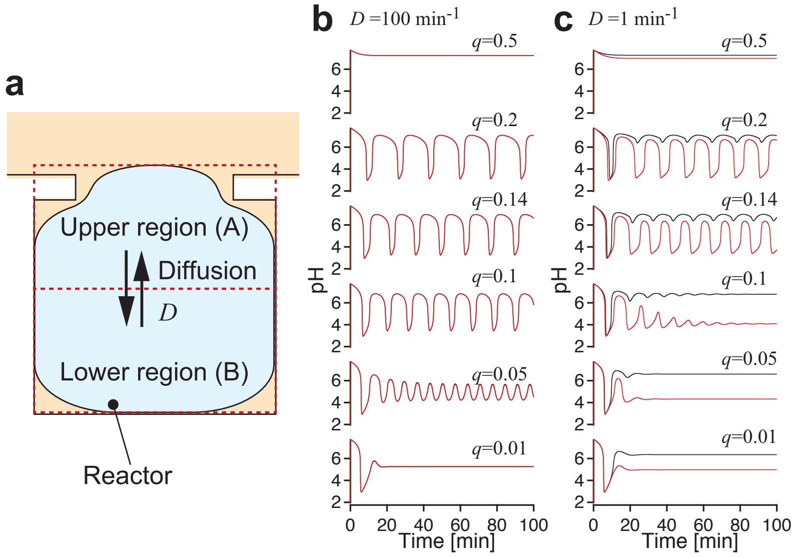

Supplementary Figure 6. Spatio-temporal analyses of droplet open-reactor system. (**a**) Simple two-region model considering diffusion of chemicals between upper and lower regions in the reactor. (**b**) and (**c**) Numerical analyses for (**b**) $D = 100$ min^{-1} and (**c**) $D = 1$ min^{-1}. Black line: upper region (A). Red line: lower region (B). Calculation parameters are the same as those in Supplementary Note 2.

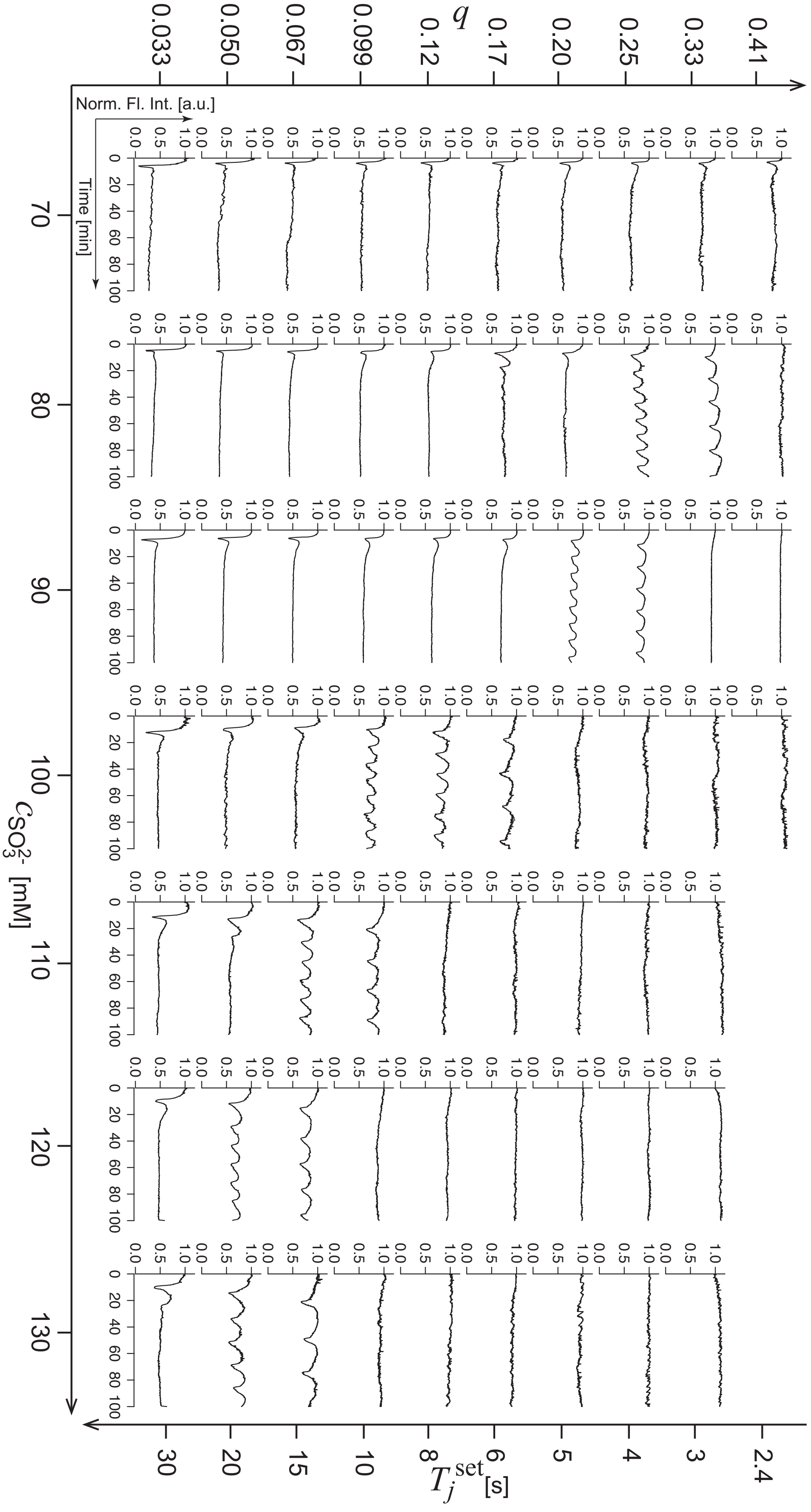

Supplementary Figure 7. Time-course data for 2D bifurcation diagram in Fig. 3f.

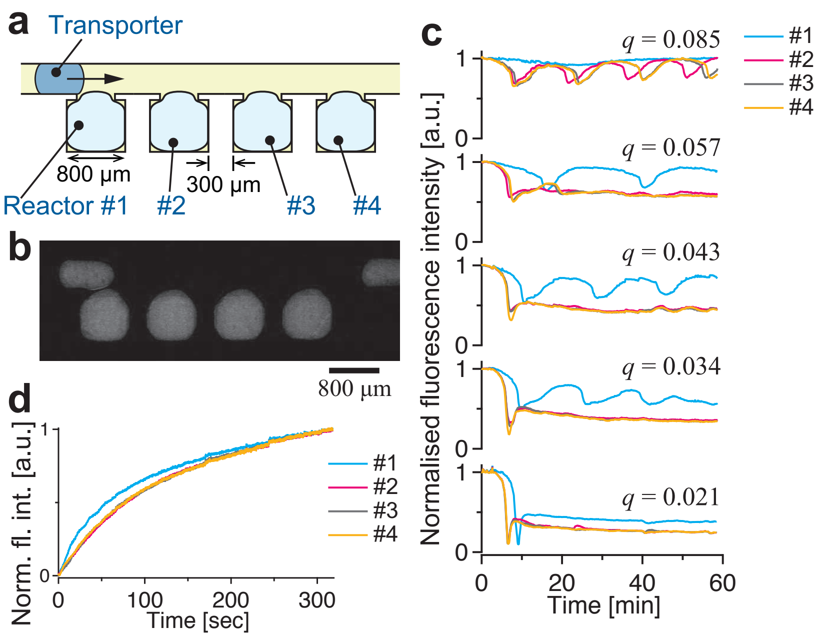

Supplementary Figure 8. Droplet open-reactor system with arrayed multiple reactors. (**a**) Design of the arrayed multiple reactors (reactors #1–#4). Except for the arrayed reactors, the microfluidic configuration is the same as that displayed in Supplementary Fig. 2a. (**b**) Fluorescent image of the arrayed multiple reactors captured during the experiment presented in (**c**). (**c**) Control of the BSF reaction in the droplet open-reactor system with arrayed multiple reactors. $F_{aq1} = F_{aq2} = 2\ \mu$L min^{-1}, and $F_{oil} = 26\ \mu$L min^{-1}. In this flow-rate, w was 0.35 s. $T = 4$ s for $q = 0.085$, $T = 6$ s for $q = 0.057$, $T = 8$ s for $q = 0.043$, $T = 10$ s for $q = 0.034$, and $T = 16$ s for $q = 0.021$. $c_{SO_3^{2-}} = 130$ mM. The concentrations of the other chemicals were the same as those described in the Methods section in the main text. The AC voltage for the droplet fusion (300 V (peak-to-peak), 1 kHz) was simultaneously applied to the four reactors using the droplet-fusion control program. (**d**) Time courses of the fluorescence intensity increase when droplet fusion occurred every ~ 2 s. The flow rates and chemical concentrations were the same as those used in the experiments (**c**). Reactor #1 exhibited a higher rate of increase than the other reactors, suggesting that the chemical fluxes of reactor #1 were greater than those of the other reactors. This difference is probably the cause of the different reaction behaviour observed in reactor #1 in (**c**).

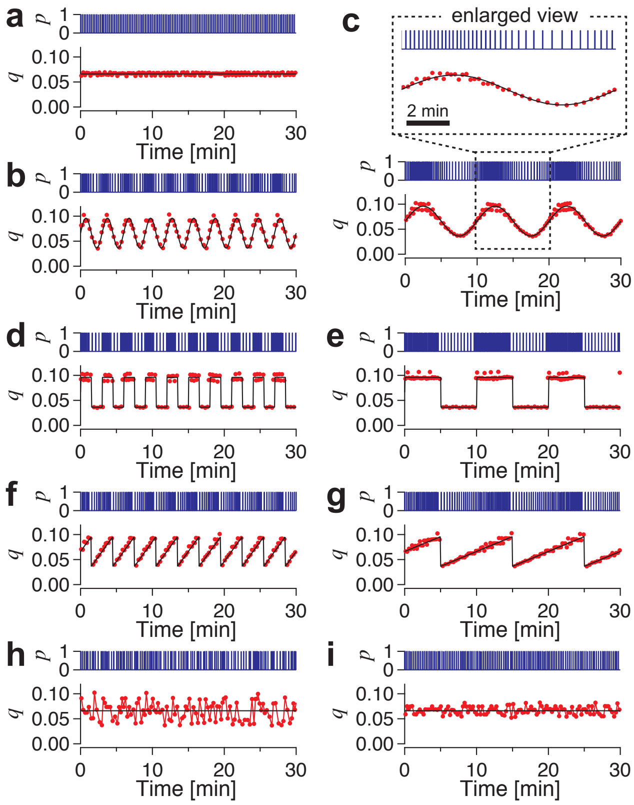

Supplementary Figure 9. Generation of p and q by pulse-density modulation control. Blue lines: pulse trains of $p(t; \mathbf{T}, \mathbf{w})$. Black lines: (**a**)–(**g**) theoretical curves, (**h**)–(**i**) theoretical averages. Red dots and lines: q generated in experiments, calculated as $q = w_j/T_j$. $w_j = 0.99$ s (fixed). (**a**) Constant function ($Z_C(t)$). (**b**) and (**c**) Sinusoidal wave functions ($Z_S(t)$, $A_q = 0.45$, $T_q = 3$ and 10 min). (**d**) and (**e**) Square wave functions ($Z_{Sq}(t)$, $A_q = 0.45$, $T_q = 3$ and 10 min). (**f**) and (**g**) Saw-tooth wave functions ($Z_{St}(t)$, $A_q = 0.45$, $T_q = 3$ and 10 min). (**h**) and (**i**) White noise functions ($Z_N(t)$, $A_q = 0.2$ and 0.45). $\overline{q} = w/\overline{T} = 0.066$ ($w = 0.99$ s, $\overline{T} = 15$ s). Solutions in the transporter and reactor were 0.1 mM fluorescein sodium. $Z_C(t)$, $Z_S(t)$, $Z_{Sq}(t)$ $Z_{St}(t)$, and $Z_N(t)$ are described in Supplementary Note 4.

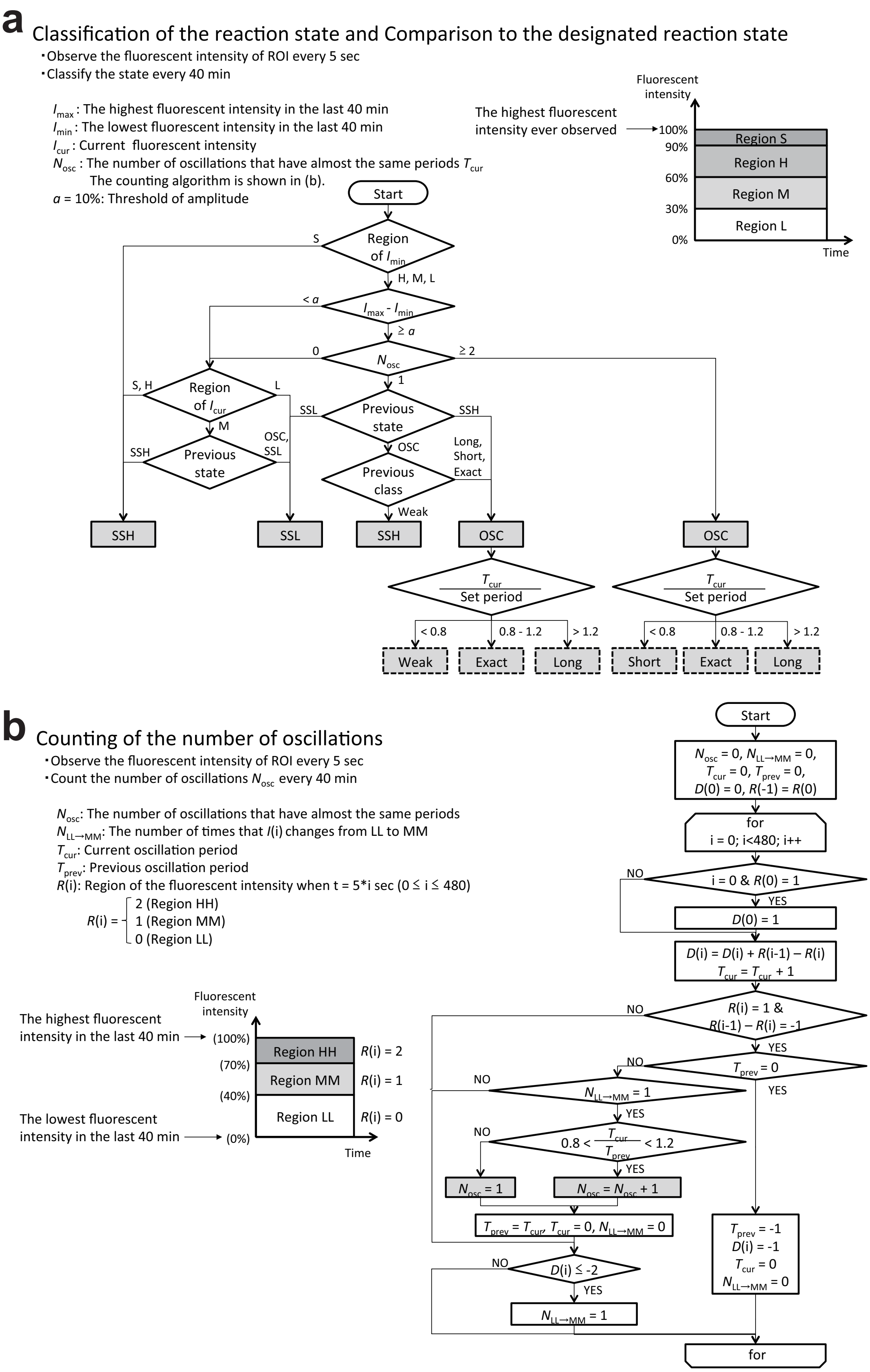

Supplementary Figure 10. Algorithm for feedback control of droplet open-reactor system; (**a**) classification of the reaction state and comparison with designated reaction state, and (**b**) counting of number of oscillations. (**b**) is the subroutine of (**a**).

Determination of the next value of *q*

- Determination of the next value of *q* after each classification of the reaction state

b = 0.0165: Pitch of the change of *q*.
q_{max}: The highest value of *q*; $q_{max}=w/T_{min}$, where T_{min} is the interval of droplet generation at the T-junction.

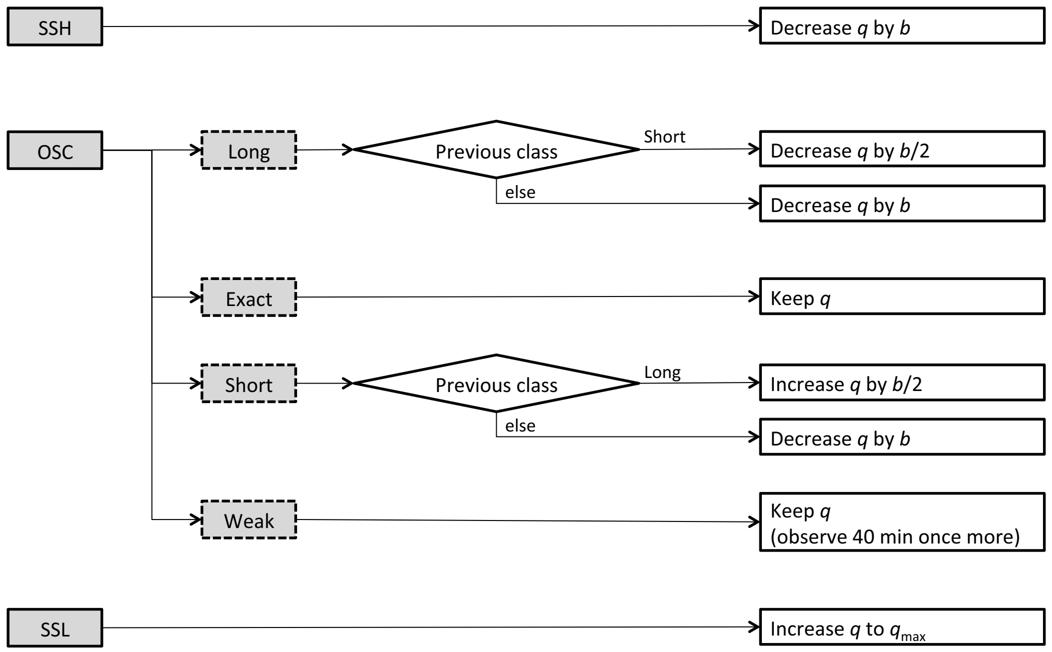

Supplementary Figure 11. Algorithm for feedback control of droplet open-reactor system; determination of next value of *q*.

Supplementary Tables

Supplementary Table 1. Kinetic reaction equations and parameters of BSF chemical reactions.

No.	Chemical reaction	Kinetic reaction equations	Kinetic parameters (ref. [3])
R1	$SO_3^{2-} + H^+ \underset{r'_1}{\overset{r_1}{\rightleftharpoons}} HSO_3^-$	$v_1 = r_1 \cdot u_{H^+} \cdot u_{SO_3^{2-}}$ (fwd)	$r_1 = 5.0 \times 10^{10}\ M^{-1}s^{-1}$
		$v'_1 = r'_1 \cdot u_{HSO_3^-}$ (bwd)	$r'_1 = 5.0 \times 10^{3}\ s^{-1}$
R2	$HSO_3^- + H^+ \underset{r'_2}{\overset{r_2}{\rightleftharpoons}} H_2SO_3$	$v_2 = r_2 \cdot u_{H^+} \cdot u_{HSO_3^-}$ (fwd)	$r_2 = 6.0 \times 10^{10}\ M^{-1}s^{-1}$
		$v'_2 = r'_2 \cdot u_{H_2SO_3}$ (bwd)	$r'_2 = 1.0 \times 10^{9}\ s^{-1}$
R3	$BrO_3^- + 3HSO_3^- \xrightarrow{r_3} 3SO_4^{2-} + Br^- + 3H^+$	$v_3 = r_3 \cdot u_{HSO_3^-} \cdot u_{BrO_3^-}$	$r_3 = 9.8 \times 10^{-2}\ M^{-1}s^{-1}$
R4	$BrO_3^- + 3H_2SO_3 \xrightarrow{r_4} 3SO_4^{2-} + Br^- + 6H^+$	$v_4 = r_4 \cdot u_{H_2SO_3} \cdot u_{BrO_3^-}$	$r_4 = 2.2 \times 10^{1}\ M^{-1}s^{-1}$
R5	$BrO_3^- + 6Fe(CN)_6^{4-} + 6H^+ \xrightarrow{r_5, r'_5} 6Fe(CN)_6^{3-} + Br^- + 3H_2O$	$v_5 = \dfrac{r_5 \cdot u_{H^+}}{r'_5 + u_{H^+}}$	$r_5 = 2.9 \times 10^{-5}\ M\,s^{-1}$
			$r'_5 = 8.0 \times 10^{-4}\ M$
R6	$SO_4^{2-} + H^+ \underset{r'_6}{\overset{r_6}{\rightleftharpoons}} HSO_4^-$	$v_6 = r_6 \cdot u_{H^+} \cdot u_{SO_4^{2-}}$ (fwd)	$r_6 = 1.0 \times 10^{11}\ M^{-1}s^{-1}$
		$v'_6 = r'_6 \cdot u_{HSO_4^-}$ (bwd)	$r'_6 = 1.0 \times 10^{9}\ s^{-1}$
R7	$H_2SO_4 \rightarrow HSO_4^- + H^+$	(completed instantaneously)	

u_i (i = H^+, SO_3^{2-}, HSO_3^-, H_2SO_3, SO_4^{2-}, HSO_4^-, BrO_3^-) indicates concentrations of chemical species i in a reactor. 'fwd' and 'bwd' indicate forward and backward reactions, respectively.

Supplementary Notes

Supplementary Note 1. Details of the analytical calculation of $p(t;\mathbf{T},\mathbf{w})$

$p(t;\mathbf{T},\mathbf{w})$ represents a time-dependent discrete process in fusion-fission. $p(t;\mathbf{T},\mathbf{w})$ is mathematically a square-wave function, which has two values: 0 (non-fusion state) or 1 (fusion state). $\mathbf{T} = \{T_j\}$ is the fusion interval between the j-th and $(j+1)$-th fusion states (Fig. 1b). $\mathbf{w} = \{w_j\}$ $(w_j < T_j)$ is the duration of the j-th fusion state. Thus, $p(t;\mathbf{T},\mathbf{w})$ is described as

$$p(t;\mathbf{T},\mathbf{w}) = \sum_{j=-\infty}^{\infty} P(t,\tau_j,\tau_j + w_j)\,, \tag{1}$$

$$P(t,t_s,t_f) \equiv H(t-t_s) - H(t-t_f)\,, \tag{2}$$

where $P(t,t_s,t_f)$ represents a single fusion which starts at time t_s and finishes at time t_f; $H(t)$ is the Heaviside unit step function: $H(t) = 0$ $(t < 0)$; $H(t) = 1$ $(t \geq 0)$; and τ_j is the time at which j-times fusion starts (i.e. $\tau_{j+1} - \tau_j = T_j$). $p(t;\mathbf{T},\mathbf{w})$ expresses an arbitrary fusion-fission process as a time-dependent function.

First, we consider a simple case in which the fusion-fission events are periodic, i.e. T_j and w_j are constant ($T_j = T$, $w_j = w$). Additionally, $T_0 = 0$ and $T_{-l} = -T_l$ are assumed for calculation simplicity. The Heaviside unit step function, $H(t)$, is expressed in Fourier integral form:

$$H(t) = \lim_{\epsilon\to+0}\int_{-\infty}^{\infty}\frac{d\xi}{2\pi i}\frac{e^{i\xi t}}{\xi - i\epsilon}\,. \tag{3}$$

Thus,

$$\begin{aligned} p(t;\mathbf{T},\mathbf{w}) &= \lim_{\epsilon\to+0}\sum_{j=-\infty}^{\infty}\int_{-\infty}^{\infty}\frac{d\xi}{2\pi i}\frac{e^{i\xi(t-\tau_j)} - e^{i\xi(t-\tau_j-w_j)}}{\xi - i\epsilon} \\ &= \lim_{\epsilon\to+0}\sum_{j=-\infty}^{\infty}\int_{-\infty}^{\infty}\frac{d\xi}{2\pi i}\frac{e^{i\xi t}(e^{-i\xi\tau_j} - e^{-i\xi(\tau_j+w_j)})}{\xi - i\epsilon}\,. \end{aligned} \tag{4}$$

The fusion-fission events are assumed to be periodic, $\tau_j = jT$; therefore,

$$\begin{aligned} \sum_{j=-\infty}^{\infty} e^{-i\xi\tau_j} &= \sum_{j=-\infty}^{\infty} e^{-i\xi jT} \\ &= 2\pi\sum_{m=-\infty}^{\infty}\delta\left(\xi T - 2\pi m\right) \\ &= \frac{2\pi}{T}\sum_{m=-\infty}^{\infty}\delta\left(\xi - \frac{2\pi}{T}m\right)\,, \end{aligned} \tag{5}$$

where $\delta(\xi)$ is the Dirac delta function. Similarly,

$$\begin{aligned} \sum_{j=-\infty}^{\infty} e^{-i\xi(\tau_j+w_j)} &= \sum_{j=-\infty}^{\infty} e^{-i\xi jT}e^{-i\xi w} \\ &= e^{-i\xi w}\times 2\pi\sum_{m=-\infty}^{\infty}\delta(\xi T - 2\pi m) \\ &= e^{-i\xi w}\frac{2\pi}{T}\sum_{m=-\infty}^{\infty}\delta\left(\xi - \frac{2\pi}{T}m\right)\,. \end{aligned} \tag{6}$$

Thus,

$$
\begin{aligned}
&p(t;\mathbf{T},\mathbf{w})\\
&=\lim_{\epsilon\to+0}\sum_{j=-\infty}^{\infty}\int_{-\infty}^{\infty}\frac{d\xi}{2\pi i}\frac{e^{i\xi t}(e^{-i\xi jT}-e^{-i\xi(jT+w)})}{\xi-i\epsilon}\\
&=\lim_{\epsilon\to+0}\int_{-\infty}^{\infty}\frac{d\xi}{2\pi i}\frac{e^{i\xi t}-e^{i\xi(t-w)}}{\xi-i\epsilon}\frac{2\pi}{T}\sum_{m=-\infty}^{\infty}\delta\left(\xi-\frac{2\pi}{T}m\right)\\
&=\frac{1}{T}\lim_{\epsilon\to+0}\sum_{m=-\infty}^{\infty}\int_{-\infty}^{\infty}d\xi\frac{e^{i\xi t}-e^{i\xi(t-w)}}{i\xi+\epsilon}\delta\left(\xi-\frac{2\pi}{T}m\right)
\end{aligned}
$$

$$
\begin{aligned}
&=\frac{1}{T}\lim_{\epsilon\to+0}\sum_{m=-\infty}^{\infty}\frac{e^{i\frac{2\pi}{T}mt}-e^{i\frac{2\pi}{T}m(t-w)}}{i\frac{2\pi}{T}m+\epsilon}\\
&=\frac{1}{T}\left[\lim_{m\to0}\frac{e^{i\frac{2\pi}{T}mt}-e^{i\frac{2\pi}{T}m(t-w)}}{i\frac{2\pi}{T}m}\right.\\
&\qquad\left.+\sum_{m=-\infty,m\neq0}^{\infty}\frac{e^{i\frac{2\pi}{T}mt}-e^{i\frac{2\pi}{T}m(t-w)}}{i\frac{2\pi}{T}m}\right]
\end{aligned}
$$

$$
\begin{aligned}
&=\frac{w}{T}+\frac{1}{T}\sum_{m=-\infty,m\neq0}^{\infty}\frac{e^{i\frac{2\pi}{T}mt}-e^{i\frac{2\pi m}{T}(t-w)}}{i\frac{2\pi}{T}m}\\
&=\frac{w}{T}+\sum_{m=1}^{\infty}\frac{2}{\pi m}\sin\left(\frac{\pi m}{T}w\right)\cos\left(\frac{2\pi m}{T}\left(t-\frac{w}{2}\right)\right). \qquad (7)
\end{aligned}
$$

Because the phase difference is not essential, we have

$$
p(t;\mathbf{T},\mathbf{w})=\frac{w}{T}+\sum_{m=1}^{\infty}\frac{2}{\pi m}\sin\left(\frac{\pi m}{T}w\right)\cos\left(\frac{2\pi m}{T}t\right). \qquad (8)
$$

w/T is the ratio of the fusion state in the fusion-fission process, i.e. the basal strength of the chemical fluxes. The second term in Supplementary Eq. 8 is a fluctuation caused by discrete fusion-fission events.

Supplementary Note 2. Chemical reactions and a numerical model of BSF pH oscillation

In general, the chemical reaction dynamics in the reactor is described as

$$\dot{u}_i = f_i(\mathbf{u}) + p(t; \mathbf{T}, \mathbf{w})\, k_i(c_i - u_i)\,, \tag{9}$$

where t is time; $\mathbf{u} = \{u_i\}$ $(i = 1, 2, \cdots)$; u_i and c_i are the concentrations of chemical substance U_i in the reactor and transporters; $f_i(\mathbf{u})$ is a reaction term expressing chemical reactions among U_i; and k_i is the exchange rate of U_i arising from its diffusion. $p(t; \mathbf{T}, \mathbf{w})$ represents an arbitrary fusion-fission process. $\mathbf{T} = \{T_j\}$ and $\mathbf{w} = \{w_j\}$ $(w_j < T_j)$. The chemical reaction dynamics in the reactor can be treated using the following time-averaged form:

$$\dot{u}_i = f_i(\mathbf{u}) + q k_i(c_i - u_i)\,. \tag{10}$$

A reaction model of the bromate-sulfite-ferrocyanide (BSF) pH oscillation reaction was developed by Edblom et al. [1]. Later, an alternative model (the RKH model; Supplementary Table 1, R1–R5) was proposed by Rábai, Kaminaga, and Hanazaki [2]. Recently, Sato et al. [3] proposed an extended RKH model (Supplementary Table 1, R1–R7). Reaction R4 is an autocatalytic H^+ production, and reaction R5 is consumption of H^+ at low pH. Reactions R4 and R5 therefore form a negative feedback loop with time delay, which can generate a pH limit cycle oscillation under the non-equilibrium open conditions at which the substrates are supplied and the products are dissipated.

For the extended RKH model (Supplementary Table 1, R1–R7), the reaction term in Supplementary Eq. 9 is as follows:

$$\begin{aligned} f_{H^+}(\mathbf{u}) = &- v_1 + v_1' - v_2 + v_2' \\ &+ 3v_3 + 6v_4 - 6v_5 - v_6 + v_6'\,, \end{aligned} \tag{11}$$

$$f_{SO_3^{2-}}(\mathbf{u}) = - v_1 + v_1'\,, \tag{12}$$

$$f_{HSO_3^-}(\mathbf{u}) = v_1 - v_1' - v_2 + v_2' - 3v_3\,, \tag{13}$$

$$f_{H_2SO_3}(\mathbf{u}) = v_2 - v_2' - 3v_4\,, \tag{14}$$

$$f_{SO_4^{2-}}(\mathbf{u}) = - v_6 + v_6' + 3v_3 + 3v_4\,, \tag{15}$$

$$f_{HSO_4^-}(\mathbf{u}) = v_6 - v_6'\,, \tag{16}$$

$$f_{BrO_3^-}(\mathbf{u}) = - v_3 - v_4 - v_5\,, \tag{17}$$

where v_1, v_1', v_2, v_2', v_3, v_4, v_5, v_6, and v_6' are given in Supplementary Table 1. The values of k_i for all chemicals are assumed to be an identical, i.e. $k = 1$ min^{-1}. In the simulations in Fig. 3e, $c_{SO_3^{2-}} = 100$ mM, $c_{HSO_3^-} = 0$ mM, $c_{H_2SO_3} = 0$ mM, $c_{BrO_3^-} = 75$ mM, and $c_{H_2SO_4} = 7.5$ mM. $\{c_i\}$ indicates the concentration of chemical species i in the transporters. Given $\gamma \equiv c_{SO_4^{2-}}/c_{H_2SO_4}$, from reactions R6 and R7 in Supplementary Table 1, we have

$$\gamma = \frac{-1 + \sqrt{1 + 4(r_6/r_6')c_{H_2SO_4}}}{2(r_6/r_6')c_{H_2SO_4}} < 1\,. \tag{18}$$

Therefore, $c_{SO_4^{2-}} = \gamma \times c_{H_2SO_4} = 5$ mM, $c_{HSO_4^-} = (1-\gamma) \times c_{H_2SO_4} = 2.5$ mM, $c_{H^+} = (1+\gamma) \times c_{H_2SO_4} = 12.5$ mM.

To obtain the 2D bifurcation diagram (Fig. 3g), we perform linear stability analysis for the time-averaged form Supplementary Eq. 10. First, we obtain a steady state of Supplementary Eq. 10, and linearise Supplementary Eq. 10 around the steady state. Secondly, a Jacobian matrix for the linearised equations is calculated. Finally, the stability of the reaction system is estimated based on the eigenvalues of the Jacobian matrix. If the real parts of all the eigenvalues are negative for a given concentration and q, the reaction system under the condition is stable, i.e. the reaction converges to the steady state. In other cases, the reaction system is unstable; the reaction under the condition therefore exhibits a limit cycle oscillation. In Fig. 3g, $c_{SO_3^{2-}}$ is variable, and the other chemical concentrations are the same as above.

Supplementary Note 3. Spatio-temporal analysis of pH oscillation in droplet open-reactor system

To estimate the effect of imperfect stirring of a reaction solution in the reactor, we analyze a simple two-region model, considering diffusion of chemicals between the upper and lower regions in the reactor, as follows (Supplementary Fig. 6a):

$$\dot{u}_i^{\mathrm{A}} = f_i(\mathbf{u}^{\mathrm{A}}) + p(t; \mathbf{T}, \mathbf{w})\, k_i(c_i - u_i^{\mathrm{A}}) + D(u_i^{\mathrm{B}} - u_i^{\mathrm{A}}), \tag{19}$$

$$\dot{u}_i^{\mathrm{B}} = f_i(\mathbf{u}^{\mathrm{B}}) + D(u_i^{\mathrm{A}} - u_i^{\mathrm{B}}), \tag{20}$$

where $\mathbf{u}^{\mathrm{A}} = \{u_i^{\mathrm{A}}\}$ and $\mathbf{u}^{\mathrm{B}} = \{u_i^{\mathrm{B}}\}$ $(i = 1, 2, \cdots, n)$ indicate the concentrations of chemical species $\{U_i\}$ in the upper (A) and lower (B) regions, respectively. D is the exchange rate of chemicals resulting from diffusion between the upper and lower regions.

When a solution in the reactor is well stirred, i.e. the exchange rate is high ($D = 100\ \mathrm{min}^{-1}$), the time courses of the pH in the upper and lower regions are approximately the same (Supplementary Fig. 6b). In contrast, when a solution in the reactor is not well stirred, i.e. the exchange rate is low ($D = 1\ \mathrm{min}^{-1}$), the time courses of the pH in the upper and lower regions behave differently (Supplementary Fig. 6c). If the spatial information is controlled more precisely using factors such as the droplet shape, this type of imperfect mixing may increase the system complexity [4].

Supplementary Note 4. Generation of p and q by pulse-density modulation control

To generate time-variable chemical fluxes, we used

$$T_j^{\mathrm{set}} = w/q(t_j), \tag{21}$$

$$q(t) = \overline{q}[1 + Z_q(t)], \tag{22}$$

where $t_0 = 0$; $t_j = \sum_{l=0}^{j-1} T_l^{\mathrm{set}}$; and $\overline{q}$ is the baseline value of $q(t)$. $Z_q(t)$ was varied according to designated functions as follows:

$$\text{Constant:} \quad Z_{\mathrm{C}}(t) = 0 \tag{23}$$

$$\text{White noise:} \quad Z_{\mathrm{N}}(t) = A_q U(-1, 1) \tag{24}$$

$$\text{Sinusoidal wave:} \quad Z_{\mathrm{S}}(t) = A_q \sin(2\pi t/T_q) \tag{25}$$

$$\text{Saw-tooth wave:} \quad Z_{\mathrm{St}}(t) = A_q[2R(t, T_q)/T_q - 1] \tag{26}$$

$$\text{Square wave:} \quad Z_{\mathrm{Sq}}(t) = A_q[2S(t, T_q) - 1] \tag{27}$$

where $U(-1, 1)$ indicates uniform random numbers between $[-1, 1]$; $R(t, T_q)$ gives the residue obtained when t is divided by T_q; and $S(t, T_q)$ gives 1 when $R(t, T_q) < T_q/2$, and otherwise gives 0.

Supplementary Figure 9 shows the generated p and q by pulse-density modulation control.

Supplementary Note 5. Fabrication of microfluidic device for droplet open-reactor system

The microfluidic system was constructed using two poly(methyl methacrylate) (PMMA) plates (1 mm thickness, extruded PMMA, Mitsubishi Rayon; Supplementary Fig. 2). The microchannel was designed using three-dimensional (3D) computer aided design (CAD) software (Rhinoceros 3D, McNeel), and was fabricated on the upper plate using a fine milling machine (MDX-40A, Roland DG) with a 0.5 mm diameter endmill (MSEM230-0.5, NS Tool). The upper plate had four holes for three inlets and an outlet, and two square holes for electrodes; additionally, it had one more hole at the top of the square chamber for injection of a reactor. The upper and bottom plates were attached by ∼ 30 min of thermal compression bonding at ∼ 90°C (AL194-L, Romanov). At the beginning of the experiments, the reactor was introduced into the square chamber through the top hole using a micropipette; the hole was then sealed with a transparent cellophane tape (Scotch 313, 3M). The oil and aqueous phases were then flowed. Details are given in Supplementary Fig. 2.

The electrodes were made of low-melting-temperature solder (SMD-B05, Sunhayato), and were connected to a function generator (WF1974, NF Corporation) through a voltage amplifier (M-2629B-2CH, MESS-TEK).

Two aqueous phases and an oil phase were introduced through inlets using ethylene tetrafluoroethylene (ETFE) tubes (Tokyo Glass Kikai) (inner diameter: 0.50 mm; outer diameter: 1.59 mm). The ETFE tubes were attached to the upper PMMA plate with an epoxide-based adhesive (AR-R30, Nichiban).

Supplementary References

1. Edblom, E., Luo, Y., Orban, M., Kustin, K. & Epstein, I. R. Systematic design of chemical oscillators. 45. Kinetics and mechanism of the oscillatory bromate-sulfite-ferrocyanide reaction. *J. Phys. Chem.* **93**, 2722–2727 (1989).

2. Rabai, G., Kaminaga, A. & Hanazaki, I. Mechanism of the Oscillatory Bromate Oxidation of Sulfite and Ferrocyanide in a CSTR. *J. Phys. Chem.* **100**, 16441–16442 (1996).

3. Sato, N., Hasegawa, H. H., Kimura, R., Mori, Y. & Okazaki, N. Analysis of the Bromate–Sulfite–Ferrocyanide pH Oscillator Using the Particle Filter: Toward the Automated Modeling of Complex Chemical Systems. *J. Phys. Chem. A* **114**, 10090–10096 (2010).

4. Epstein, I. R. The consequences of imperfect mixing in autocatalytic chemical and biological systems. *Nature* **374**, 321–327 (1995).

5. Takinoue, M., Onoe, H. & Takeuchi, S. Fusion and fission control of picoliter-sized microdroplets for changing the solution concentration of microreactors. *Small* **6**, 2374–2377 (2010).
